# The impact of acute blood‐flow‐restriction resistance exercise on somatosensory‐evoked potentials in healthy adults

**DOI:** 10.1113/EP091911

**Published:** 2024-09-04

**Authors:** Tom Maudrich, Sebastian Degener, Patrick Ragert, Rouven Kenville

**Affiliations:** ^1^ Department of Movement Neuroscience, Faculty of Sports Science Leipzig University Leipzig Germany; ^2^ Department of Neurology Max Planck Institute for Human Cognitive and Brain Sciences Leipzig Germany

**Keywords:** biceps curl, blood‐flow restriction, somatosensory‐evoked potentials, somatosensory excitability

## Abstract

Blood‐flow‐restriction exercise (BFR_EX_) is an emerging method to stimulate hypertrophy and strength without the need for high training loads. However, the impact of BFR_EX_ concerning somatosensory processing remains elusive. Here, we aimed to investigate the acute effects of BFR_EX_ on somatosensory processing in healthy adults using somatosensory‐evoked potentials (SEPs). Twelve healthy adults (23.0 ± 3.2 years of age) participated in a randomized crossover experiment, consisting of three experimental conditions: application of blood‐flow restriction without resistance exercise (BFR), resistance exercise for multiple sets with blood‐flow restriction (BFR_EX_) and traditional resistance exercise (unilateral biceps curls) for multiple sets without BFR (EX). SEP measurements were recorded bilaterally before, during and after each condition. SEP amplitudes were largely unaffected during various occlusive conditions. Nonetheless, our findings demonstrate a significant decrease in N9 latencies for condition EX compared with BFR, specifically in the exercised limb (mean difference = −0.26 ms, SE = 0.06 ms, *P* = 0.002, *d* = −0.335). This study provides evidence on the lack of impact of BFR_EX_ within the somatosensory domain, according to current guidelines. As an alternative method to traditional high‐load resistance exercise, BFR_EX_ might offer a considerable upside for rehabilitative settings by reducing strain on the musculoskeletal system.

## Abstract (highlights)

- **What is the central question of this study?**
  Blood‐flow‐restriction exercise (BFR_EX_) is a low‐load alternative to traditional resistance exercise for rehabilitative settings. Crucially, the impact of BFR_EX_ concerning somatosensory processing remains elusive.
- **What is the main finding and its importance?**
  Here, we demonstrate that somatosensory processing, as assessed by somatosensory‐evoked potentials, is largely unaffected by acute BFR_EX_ in healthy adults adhering to contemporary guidelines, highlighting its potential application in rehabilitative settings.

## 1 INTRODUCTION

Targeted resistance exercise is a fundamental component in numerous sports, aiming to enhance athletic performance through hypertrophy and increased muscle strength. A well‐developed musculoskeletal system offers substantial benefits across all age groups, making resistance exercise also integral to rehabilitative processes. Resistance exercise promises considerable benefits, especially in neurorehabilitation (Harris & Eng, 2010). At its essence, this approach focuses on enhancing muscle strength, directly addressing the weakened musculature at the core of many neurological conditions (Bohannon, 2007). Prior research has underscored the capacity of resistance exercise to support neurorehabilitation efforts. However, caution is often exercised in its implementation owing to concerns about the mechanical strain it imposes on the body, potentially diminishing its efficacy (Harris & Eng, 2010). Consequently, exploring alternative methods to execute resistance exercises with reduced strain while maximizing their effectiveness in promoting muscle hypertrophy and enhancing strength becomes imperative.

One emerging method to stimulate hypertrophy and strength without the need for high training loads is blood‐flow‐restriction exercise (BFR_EX_). This approach allows significant strength and muscle mass gains with low training loads (Patterson et al., 2019). Current research indicates that chronic BFR_EX_ and conventional resistance exercise can yield similar results in terms of muscle growth and strength gains, although strength gains are commonly more pronounced in high‐load resistance exercise protocols (Rodrigo‐Mallorca et al., 2021). A clear advantage of BFR_EX_ is the reduced external load, as low as 15% of one‐repetition maximum (1‐RM) (Jessee et al., 2018) compared with 70%–85% of 1‐RM for traditional resistance exercise, reducing strain on both active and passive musculoskeletal systems. Consequently, individuals previously limited by injuries or other constraints are thus able to engage in effective resistance exercise (Lixandrao et al., 2018). In BFR_EX_, the proximal application of a tourniquet cuff restricts arterial inflow and venous outflow in the target muscle. This leads to oxygen deprivation within the muscle during exercise, which, in turn, induces metabolic stress (Loenneke et al., 2011). Combined with mechanical tension on the muscles during exercise, these two mechanisms are believed primarily to cause the significant hypertrophy effects observed following BFR_EX_ (Pearson & Hussain, 2015).

As is typical of modern methods with potentially wide‐ranging applications, there is considerable interest in investigating the safety and associated physiological effects of BFR_EX_ (Nascimento et al., 2022). Although BFR_EX_ has been thoroughly investigated concerning cardiovascular responses (Patterson et al., 2019), the physiological effects of acute and chronic BFR_EX_ on central and peripheral neural mechanisms remain understudied (Centner & Lauber, 2020). Consequently, neuromuscular functioning following BFR_EX_ has received limited attention in existing research. This is, however, an important consideration, because pronounced limb compression could potentially lead to impaired nerve function (Thatte & Mansukhani, 2011). Several studies have examined the effects of low‐intensity BFR_EX_ on peripheral nerve function, finding no negative impact on nerve conduction properties (Clark et al., 2011; Mendonca et al., 2020), categorizing BFR_EX_ as a neurologically harmless training method. Another understudied aspect of neuromuscular functioning concerns the influence of BFR_EX_ on the excitability of the somatosensory system. In particular, the study of somatosensory evoked potentials (SEPs) offers additional insights into the characteristics of neural responses at different levels of the neural pathway. Here, electrical stimulation of peripheral nerves, such as the median and tibial nerves, is used to assess evoked responses in the upper and lower limbs, respectively. Far‐field potentials, which are generated far away from the recording site on the scalp, reflect the excitation of peripheral nerve cells in the spinal cord and subcortical structures (Ghigo et al., 1991). In contrast, near‐field potentials are generated near the scalp recording electrodes in the cortex. Short‐latency cortical SEPs following upper limb stimulation have peak latencies between 18 and 35 ms, with the initial cortical response, known as the N20 component, observed in parietal regions (Yamada, 1988). Somatosensory evoked potentials are traditionally used in clinical settings to assess the integrity of the peripheral and central nervous system (Walsh et al., 2005). In addition, SEPs have been implemented in sports science research to investigate training‐induced neuroplasticity, somatosensory excitability and athletic expertise (Maudrich et al., 2022). Regarding BFR_EX_, an initial study by Hayashi et al. (2019) revealed that deafferentation of the median nerve through ischaemic nerve blockade resulted in reduced responses to SEPs for N9 and N20, which were entirely suppressed over time. Upon resolution of deafferentation, an overactivation of the sensory cortex occurred. Arguably, with BFR_EX_, using a proximally applied cuff and consequent attenuated compression of the target limb, resembling partial deafferentation, similar modulations of SEP components might be observed. Accumulation of metabolites and blood distal to the applied cuff could lead to an increase in occlusion pressure during exercise, potentially amplifying the effects of partial deafferentation in comparison to the sole use of a cuff. Another aspect that might additionally influence SEP modulation concerns the perceived pain during BFR_EX_, because the accumulation of metabolic stress is often associated with discomfort or pain (Pollak et al., 2014). Various factors can influence pain sensation, with proximity to muscle failure having a significant impact, because maximal metabolic stress is reached at that point (Spitz et al., 2022). This could also influence the excitability of the somatosensory system, because severe discomfort might modulate afferent signals, thereby affecting the willingness of an individual to exert effort during performance (Hureau et al., 2018). Notably, a recent study demonstrated decreased pain sensation in participants engaging in BFR_EX_ in comparison to those performing traditional high‐load resistance exercise (Early et al., 2020).

With the present study, we, therefore, aimed to investigate the acute effects of BFR_EX_ on the excitability of the somatosensory system of healthy adults. The BFR_EX_ was performed using unilateral upper extremity resistance exercise, and SEP analyses were performed bilaterally not only before and during the application of BFR_EX_, but also after pressure release. Based on the outlined research, we hypothesized to observe a suppression of relevant short‐latency SEP amplitudes and prolonged latencies during BFR_EX_. These findings will allow us to gain a better understanding of the practical implications of potential BFR_EX_ modulations, which, in turn, will provide insight to make informed judgments about the practical implementation of this training approach with respect to neuromuscular functioning in clinical and rehabilitative populations.

## 2 METHODS

### 2.1 Ethical approval

The study was approved by the ethics committee of Leipzig University (ref. no. 398/22‐ek). After being made aware of the objectives, methods, potential risks and benefits of the study, participants were required to sign an informed consent form in accordance with the *Declaration of Helsinki*.

### 2.2 Participants

An a priori power analysis was performed based on previous work regarding deafferentation‐induced modulations of SEP parameters (i.e., N20) (Hayashi et al., 2019). A power value (probability of correctly rejecting a false null hypothesis) of 0.95 was chosen, given a type I error rate α = 5% and an effect size of 0.41. The estimated minimal sample size to obtain sufficient test power was *n* = 9. In total, 12 healthy adults (four female; for a detailed overview, please see Table 1) participated in this study. All participants were free of neurological disease as determined by a thorough neurological examination. Any participants with potential contraindication to BFR_EX_ (i.e., coronavirus disease 2019 infection, diabetes mellitus, hypertension, chronic kidney disease, cardiovascular disease, consumption of anabolic steroids and ergogenic substances, rheumatoid arthritis or pregnancy) were excluded from the study (Nascimento et al., 2022).

**TABLE 1:** Anthropometric and demographic data of the sample (values are expressed as the mean ± SD).

Variable	Value
Sample size	*n* = 12
Age (years)	23.0 ± 3.2
Handedness (right/left)	11/1
Height (cm)	175.8 ± 10.4
Body mass (kg)	72.8 ± 16.6
Resistance exercise experience (years)	5.0 ± 4.2
Training per week (h)	7.8 ± 3.3
Unilateral biceps curl three‐repetition maximum (kg)	14.8 ± 6.6

### 2.3 Procedures

**FIGURE 1:**
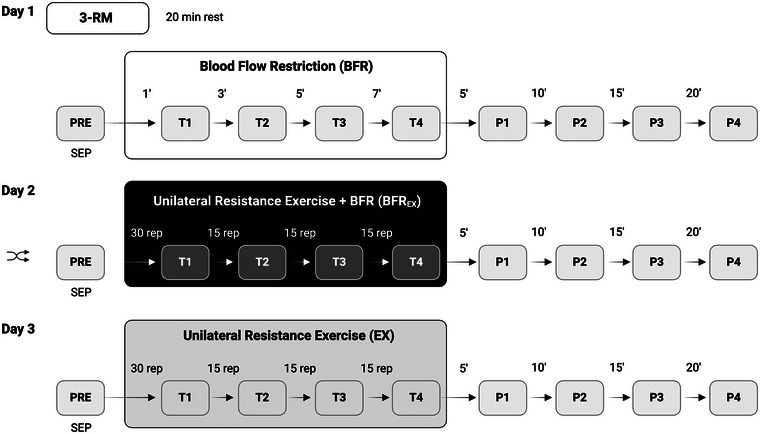
Experimental procedure. The experiment consisted of a randomized and counterbalanced cross‐over design. Each participant took part in three experimental sessions spaced by ≥3 days. During each of the experimental sessions, participants underwent one of three conditions: application of BFR without resistance exercise (BFR), resistance exercise for multiple sets with BFR (BFR_EX_) or resistance exercise (unilateral biceps curls) for multiple sets without BFR (EX). During the first experimental session, participants performed a three‐repetition maximum (3‐RM) test of the unilateral biceps curl to determine individual loads during resistance exercise followed by the condition BFR. The order of experimental days 2 and 3 (BFR_EX_ and EX) was randomized for each participant. A total of nine somatosensory evoked potential measurements were carried out during each experimental session. Abbreviation: rep, repetition; 3‐RM, three‐repetition maximum; SEP, somatosensory‐evoked potential.

This experiment consisted of a randomized and counterbalanced cross‐over design. Each participant took part in three experimental sessions spaced by ≥3 days to avoid possible impacts of neuromuscular and cognitive fatigue. During each of the experimental sessions, participants underwent one of three conditions (see Figure 1): application of BFR without resistance exercise (BFR); resistance exercise for multiple sets with BFR (BFR_EX_); or resistance exercise (unilateral biceps curls) for multiple sets without BFR (EX). Before the start of the experiment, participants were randomly assigned to perform unilateral biceps curls with the right or left arm during all experimental sessions (ACTIVE). This order was counterbalanced and independent of handedness. The opposite arm (PASSIVE) served as the intra‐individual control limb during neurophysiological measurements. Before and immediately after each experimental sessions (BFR, BFR_EX_ and EX), participants rated their level of attention, fatigue and discomfort on visual analog scales (VAS) ranging from 1 to 10. Additionally, during BFR and BFR_EX_, participants were asked to rate the discomfort of cuff pressure on a VAS scale ranging from 1 to 10.

#### 2.3.1 Experimental session 1

During the first experimental session, participants performed a three‐repetition maximum (3‐RM) test of the unilateral biceps curl to determine individual loads during unilateral resistance exercise. Instead of a 1‐RM test, a 3‐RM test was used to reduce the risk of injury during movement execution. Initially, a submaximal 5 min warm‐up was performed by each participant on a rowing ergometer. For the 3‐RM testing, successive attempts of unilateral biceps curls using dumbbells with increasing load were performed until a load was reached that could only be lifted three times by the participants with clean form. This load was defined as the individual 3‐RM. During the performance of unilateral biceps curls, participants were standing with their backs towards a wall and their elbows fixated to avoid misalignment of the upper arms and the use of momentum. Between sets with incrementally higher loads, a rest period of 3 min was granted. Once three clean repetitions of a load level were completed, the load was increased in the next attempt. If participants were not able to complete three clean repetitions of a load level, the previous load level was assumed to be 3‐RM. After 3‐RM determination (mean, 14.8 ± 6.6 kg), participants were allowed to rest for 20 min to reduce potential effects of fatigue. Individual 3‐RM values were used to determine loads for EX and BFR_EX_. Therefore, 1‐RM values were extrapolated from 3‐RM values according to a commonly used procedure (Baechle & Earle, 2008). Finally, 30% of 1‐RM was used as individual loads during resistance exercise. This load was chosen because it corresponds to the typical loads used during resistance exercise with BFR (Patterson et al., 2019).

After 3‐RM testing, participants were seated and prepared for SEP measurements (see Section 2.4) and the application of BFR. One BFR cuff (The Occlusion Cuff Pro, cuff width, 8 cm) was attached to the upper arm being trained (ACTIVE) to the most proximal region immediately below the deltoideus muscle. To standardize cuff pressure during BFR, individual arterial occlusion pressure (AOP; i.e., the amount of pressure required to cease blood flow to a limb) was determined by use of Doppler ultrasound (The Occlusion Cuff Pro Doppler). Cuff pressure used during BFR application was subsequently standardized at 50% AOP, again corresponding to contemporary guidelines (Patterson et al., 2019). Both arms, ACTIVE and PASSIVE, rested on chair rests at right angles. Next, baseline SEP measurements (PRE) were conducted bilaterally without pressure inflation. Afterwards, the pressure was applied stepwise until it corresponded to 50% AOP. Measurements of SEPs were conducted at four additional time points (T1–T4), each separated by 1 min of rest. Immediately after T4, cuff pressure was deflated and a further four SEP post measurements (P1–P4) were conducted spaced by 5 min of rest to assess the effects of BFR. During resting phases, movements were prohibited to avoid differences in excitability between participants. A total of nine SEP measurements were carried out during BFR. In total, experimental session 1 lasted ∼120 min. A general overview of the procedure can be seen in Figure 1.

#### 2.3.2 Experimental sessions 2 and 3

Both experimental sessions 2 and 3 followed a similar procedure. Participants were again seated with both arms (ACTIVE and PASSIVE) resting on chair rests at right angles and prepared for SEP and EMG measurements. For BFR_EX_, one occlusion cuff was attached to ACTIVE, and AOP was determined in the same way as during experimental session 1. Next, baseline SEP (PRE) was measured; thereafter, the cuff was inflated with 50% AOP for unilateral resistance exercise with BFR. Here, four sets with 30% of 1‐RM of unilateral biceps curls were performed by the participant (repetition scheme: 30–15–15–15) following contemporary guidelines (Patterson et al., 2019). Repetition tempo was standardized at 2 s eccentric and 2 s concentric movements, which was assured by one of the researchers. Overall, 2 of 12 participants achieved concentric muscular failure while performing BFR_EX_ during the third of four sets at repetition 11 and 12, respectively. The participants were then asked to achieve the targeted number of repetitions with lengthened partial movement execution. To account for this, training loads for the last set were then individually reduced by 20% for both participants. With this adjustment, both participants were able to complete the desired number of 15 repetitions during the last set. Immediately after each set, bilateral SEP was measured without releasing occlusion pressure (T1–T4). Therefore, participants were instructed to relax and prepare for the next SEP measurement as quickly as possible after the last repetitions of each set. After T4, occlusion pressure was released, and four more SEP measurements were acquired with 5 min of rest in between (P1–P4).

The last experimental day followed the same procedure as experimental day 2. Here, the same unilateral resistance exercise was performed, but without the application of the occlusion cuff and BFR (EX). Again, four sets with 30% of 1‐RM of unilateral biceps curls were performed by the participant (repetitions scheme: 30–15–15–15) with a 2 s eccentric and 2 s concentric repetition tempo. During EX, all participants were able to perform the target number of repetitions in every set. Time points for SEP measurements corresponded to experimental day 2 (PRE, T1–T4 and P1–P4).

To avoid potential effects of fatigue after 3‐RM testing on the subsequent performance of unilateral biceps curls, BFR was always performed by participants on experimental day 1, where the cuff and occlusion pressure were applied but no resistance exercise was performed. However, the order of experimental days 2 and 3 (BFR_EX_ and EX) was randomized for each participant, with both sessions lasting ∼90 min.

### 2.4 Somatosensory‐evoked potentials

**FIGURE 2:**
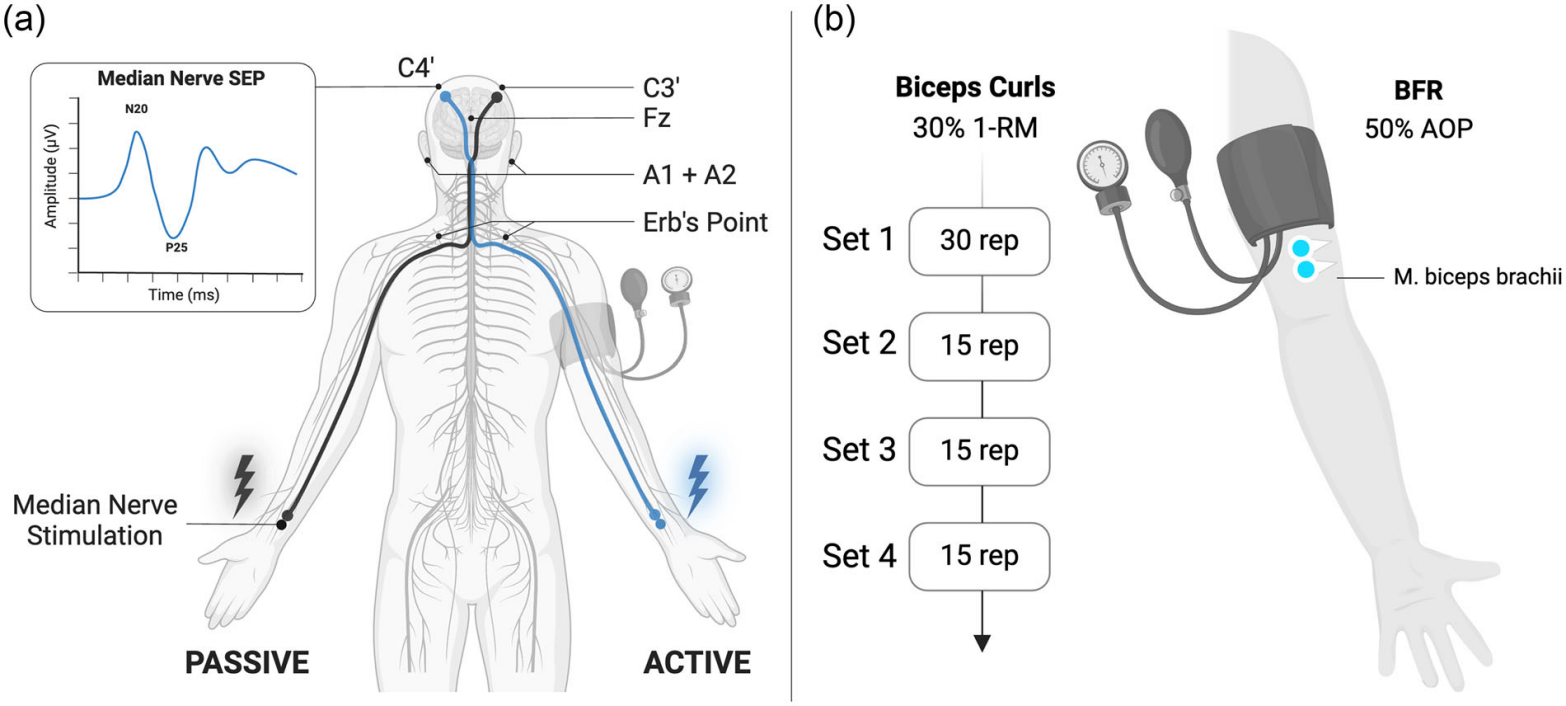
Experimental set‐up. (a) EEG recording electrodes for somatosensory evoked potential (SEP) measurements were placed at bilateral Erb's point (ERB1 and ERB2), bilateral ear lobes (A1 and A2), Fz, C3′ (2 cm behind C3) and C4′ (2 cm behind C4) according to the international 10–20 system. For each SEP measurement, bilateral median nerve stimulation was applied. One blood‐flow restriction (BFR) cuff was attached to the upper arm being trained (ACTIVE) to the most proximal region immediately below the deltoideus muscle. To standardize cuff pressure during BFR, individual arterial occlusion pressure (AOP) was determined by the use of Doppler ultrasound. Cuff pressure used during BFR application was subsequently standardized at 50% AOP. (b) Unilateral biceps curls were performed using 30% of one‐repetition maximum and according to the repetition (rep) scheme: 30–15–15–15. Repetition tempo was standardized at 2 s eccentric and 2 s concentric movements, which was assured by one of the researchers. During the performance of unilateral biceps curls, surface EMG activity of the biceps brachii (BB) muscle was recorded unilaterally from ACTIVE. Abbreviation: 1‐RM, one‐repetition maximum.

Measurements and analysis of SEPs were performed with a combined stimulation and EEG recording device (Neuropack X1, Nihon Kohden, Tokyo, Japan). The scalp of the participants, the earlobes and the supraclavicular fossae were prepared by cleaning with an abrasive paste (OneStep AbrasivePlus Gel). EEG recording electrodes were placed at bilateral Erb's point (ERB1 and ERB2), bilateral ear lobes (A1 and A2), Fz, C3′ (2 cm behind C3) and C4′ (2 cm behind C4) according to the international 10–20 system (see Figure 2a). The recording montage for right median nerve stimulation was as follows: N9, right Erb's point–(A1 + A2); P14, C3′–(A1 + A2); and N20, C3′–Fz. The opposite montage was used for left median nerve stimulation. According to International Federation of Clinical Neurophysiology (IFCN) guidelines (Cruccu et al., 2008), putative generators of N9 are thought to represent the proximal peripheral volley from the brachial plexus around Erb's point, P14 recorded as the far‐field potential from the scalp is assumed to be generated at or near the first synaptic relay of the lemniscal system in the cuneate nucleus, and N20 is considered to be generated by neurons in the anterior wall of the postcentral gyrus (S1), Brodmann area 3b contralateral to stimulation. Electrode impedance was standardized and maintained at <5 kΩ. An earth electrode was attached to the inactive arm (PASSIVE). The SEP responses were digitized at a sample rate of 5120 Hz with an online bandpass filter set at 5–1500 Hz. For each SEP measurement, bilateral median nerve stimulation with 300 square‐wave pulses (0.2 ms) at a stimulation frequency of 5 Hz was applied. Stimulation electrodes were attached bilaterally at the wrist using Velcro tape to assure identical stimulation sites during repeated SEP measurements. Stimulus intensity was set individually at motor threshold plus 2 mA and kept constant for all SEP measurements of an experimental session. In total, nine SEP measurements were conducted during each experimental session (see Figure 1). Averaged SEP traces of each measurement were used to assess the latencies of N9, P14 and N20 peaks. Furthermore, peak‐to‐peak amplitudes for components P8–N9, P14–N20 and N20–P25 were determined. Before further statistical analyses, amplitudes of all components were normalized to values obtained at baseline (PRE).

### 2.5 Electromyography

During BFR_EX_ and EX, muscle activity of the biceps brachii (BB) muscle was recorded from ACTIVE (see Figure 2b) using a wireless Ultium EMG telemetry system (Noraxon, Scottsdale, AZ, USA). Optimal signal quality during recording was assured through skin preparation (i.e., abrasion and cleaning with alcohol). Gel‐coated self‐adhesive surface electrodes (interelectrode distance of 20 mm) were mounted on a standardized electrode position according to SENIAM recommendations (Hermens et al., 2000), distally to the occlusion cuff. Based on anatomical landmarks, electrode placements were kept constant between experimental sessions. EMG electrodes were positioned in parallel to the direction of the muscle fibres. Data were recorded with a sampling rate of 2000 Hz with an online bandpass filter in the frequency range of 10–500 Hz. Before the beginning of each experimental session, maximum voluntary contraction (MVC) values were determined to enable normalization of EMG activity. To determine the MVC of BB, three maximal isometric contractions (5 s) were performed in standardized position with the upper arm flexed at 90° and the elbow placed on a table in front of the participants. Between each MVC trial, a 30 s rest period was granted. EMG amplitudes of BB were computed from the EMG signal recorded during BFR_EX_ and EX using myoRESEARCH software (MR3.12, Noraxon). Therefore, muscle on‐ and off‐sets of each contraction were determined visually by a single trained researcher. Next, signals were full‐wave rectified, and root mean square values (100 ms) were obtained. Furthermore, mean amplitudes for each contraction were calculated (75 contractions per experimental session). Before further analysis, all EMG amplitudes were normalized to individual MVC values recorded at the beginning of each experimental session using the highest EMG amplitude of all three MVCs of each participant. Last, MVC‐normalized EMG amplitudes were averaged set‐wise (SET1–SET4) for each experimental session separately before statistical analysis.

### 2.6 Statistical analysis

All statistical analyses were carried out using JASP v.0.18.1 (University of Amsterdam, Amsterdam, The Netherlands). The majority of amplitudes and latencies of all SEP components were normally distributed as assessed through Shapiro–Wilk testing (α = 0.05). Furthermore, AOP values, SEP stimulation intensities and MVC‐normalized EMG amplitudes were normally distributed.

The VAS were analysed for each scale (attention, fatigue and discomfort) using separate repeated‐measures ANOVAs with the within‐subject factors of CONDITION (BFR, BFR_EX_ and EX) and TIME (PRE and POST). The VAS of pressure discomfort during BFR and BFR_EX_ was assessed using Student's paired *t*‐test. Effect sizes of Student's paired *t*‐test were reported as Cohen's *d*.

Differences in AOP values between experimental sessions BFR and BFR_EX_ were assessed using Student's paired *t*‐test.

Stimulation intensities of SEP measurements were compared using a repeated‐measures ANOVA with the within‐subject factors of CONDITION (BFR, BFR_EX_ and EX) and HAND (ACTIVE and PASSIVE).

Amplitudes (P8–N9, P14–N20 and N20–P25) and latencies (N9, P14 and N20) of SEP components were analysed using separate repeated‐measures ANOVAs with the within‐subject factors LIMB (ACTIVE and PASSIVE), CONDITION (BFR, BFR_EX_ and EX) and TIME (PRE, T1–T4 and P1–P4) to test for differences between limbs and experimental sessions.

Last, MVC‐normalized EMG amplitudes were compared between experimental sessions using a repeated‐measures ANOVA with the within‐subject factors CONDITION (BFR_EX_ and EX) and TIME (SET1–SET4). Pooled EMG amplitudes of SET1–SET4 were compared using Student's paired *t*‐test.

Sphericity violations of ANOVAs were addressed through Greenhouse–Geisser correction. Effect sizes are reported using η_p_^2^. *Post hoc* analyses were conducted with Bonferroni–Holm correction for multiple comparisons, and effect sizes were reported as Cohen's *d*. For all statistical analyses, a *P*‐value of *P* < 0.05 was considered significant.

## 3 RESULTS

The VAS showed no differences between experimental sessions (attention: *F*_2,22_ = 0.358, *P* = 0.703, η_p_^2^ = 0.032; fatigue: *F*_2,22_ = 0.128, *P* = 0.880, η_p_^2^ = 0.012; discomfort: *F*_1.26,13.82_ = 2.563, *P* = 0.127, η_p_^2^ = 0.189) and no significant changes from PRE to POST (attention: *F*_1,11_ = 1.453, *P* = 0.253, η_p_^2^ = 0.117; fatigue: *F*_1,11_ = 0.178, *P* = 0.681, η_p_^2^ = 0.016; discomfort: *F*_1,11_ = 0.789, *P* = 0.393, η_p_^2^ = 0.067). Furthermore, no significant interaction in CONDITION × TIME was found (attention: *F*_2,22_ = 2.962, *P* = 0.073, η_p_^2^ = 0.212; fatigue: *F*_2,22_ = 1.870, *P* = 0.178, η_p_^2^ = 0.145; discomfort: *F*_1.06,11.69_ = 1.331, *P* = 0.275, η_p_^2^ = 0.108).

The level of pressure discomfort showed no significant difference between BFR and BFR_EX_ [mean difference (MD) = −1.167, *t*_11_ = −1.984, *P* = 0.073, *d* = −0.573], with BFR_EX_ tending to cause more discomfort.

Furthermore, AOP values did not differ between BFR and BFR_EX_ (MD = −2.417 mmHg, *t*_11_ = −0.717, *P* = 0.488, *d* = −0.207).

No differences in stimulation intensity of SEP measurements were found between experimental sessions (*F*_1.296,14.252_ = 0.469, *P* = 0.553, η_p_^2^ = 0.041) or between ACTIVE and PASSIVE (*F*_1,11_ = 0.009, *P* = 0.926, η_p_^2^ > 0.001). Moreover, no interaction effect in CONDITION × HAND was found (*F*_2,22_ = 0.630, *P* = 0.542, η_p_^2^ = 0.054).

### 3.1 Amplitude of SEPs

**FIGURE 3:**
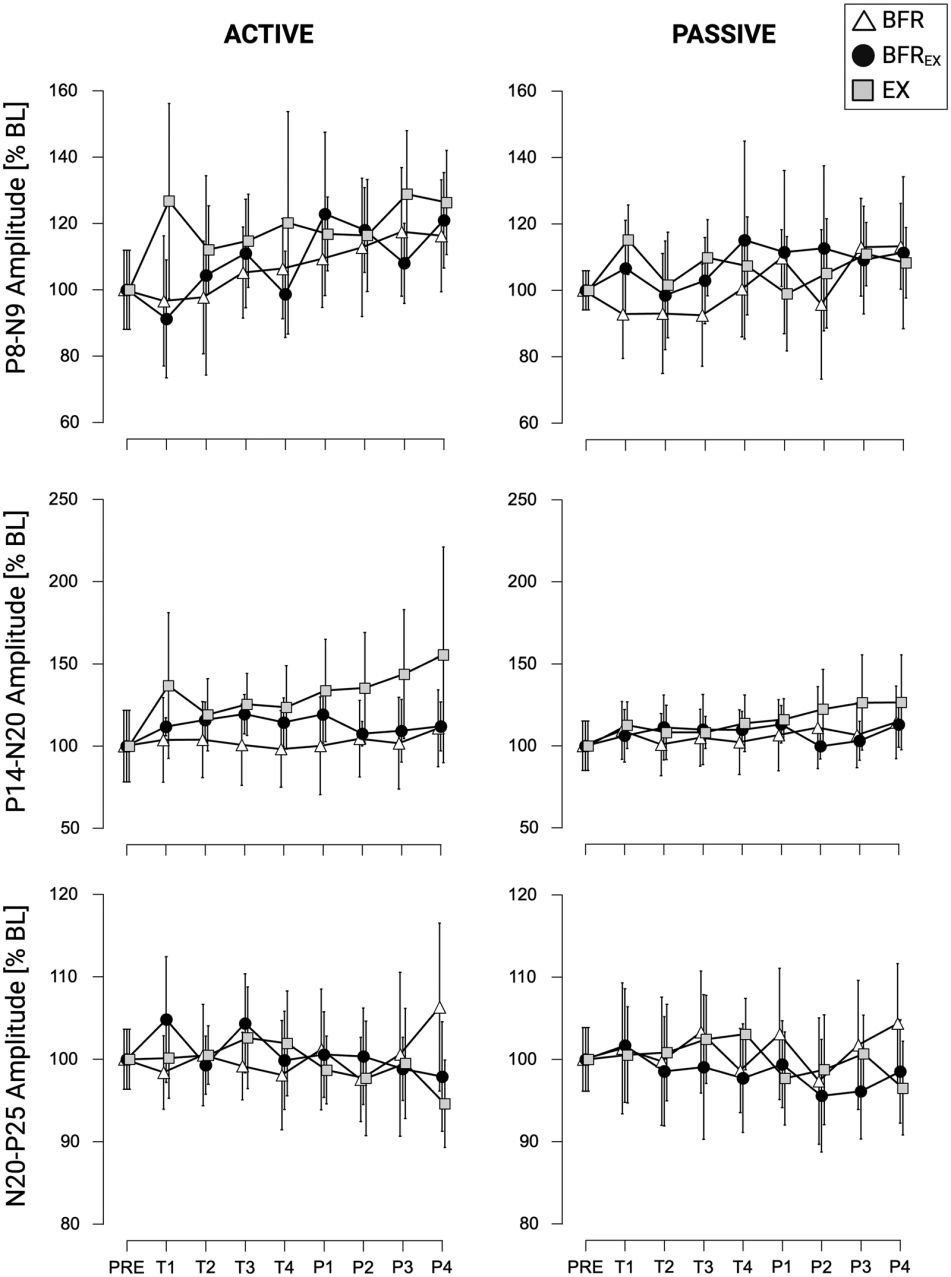
Mean somatosensory evoked potential (SEP) amplitudes (P8–N9, P14–N20 and N20–P25) for ACTIVE and PASSIVE normalized to baseline (PRE). Mean values are indicated; the error bars represent 95% confidence intervals (*n* = 12 per condition). All individual responses can be found in the raw dataset provided. Abbreviation: BL, baseline.

Repeated‐measures ANOVA indicated a significant effect for the factor TIME on P8–N9 peak amplitudes F_2.492,27.414_ = 3.671, *P* = 0.030, η_p_^2^ = 0.250). Post‐hoc analyses revealed that amplitudes were larger during P3 (MD = 14.5%, SE = 4.1%, *P* = 0.022, *d* = 0.480) and P4 (MD = 16.1%, SE = 4.1%, *P* = 0.007, *d* = 0.530) when compared with PRE. Furthermore, amplitudes were larger during P4 compared with T2 (MD = 14.9%, SE = 4.1%, *P* = 0.018, *d* = 0.490) (see Figure 3).

However, no other significant effect for LIMB, TIME or CONDITION and no significant LIMB × CONDITION or LIMB × CONDITION × TIME interaction was found for P8–N9, P14–N20 and N20–P25. For exact statistics, please see Table 2.

**TABLE 2:** Results of repeated‐measures ANOVA investigating somatosensory evoked potential amplitudes for the factors LIMB, CONDITION and TIME and the interactions of LIMB × CONDITION and LIMB × CONDITION × TIME.

Component	LIMB	CONDITION	TIME	LIMB × CONDITION	LIMB × CONDITION × TIME
P8–N9	*P* = 0.114 η_p_^2^ = 0.212	*P* = 0.532 η_p_^2^ = 0.056	*P* = 0.030^*^ η_p_^2^ = 0.250	*P* = 0.560 η_p_^2^ = 0.051	*P* = 0.413 η_p_^2^ = 0.087
P14–N20	*P* = 0.149 η_p_^2^ = 0.180	*P* = 0.316 η_p_^2^ = 0.093	*P* = 0.152 η_p_^2^ = 0.162	*P* = 0.254 η_p_^2^ = 0.118	*P* = 0.358 η_p_^2^ = 0.091
N20–P25	*P* = 0.871 η_p_^2^ = 0.003	*P* = 0.871 η_p_^2^ = 0.013	*P* = 0.573 η_p_^2^ = 0.060	*P* = 0.501 η_p_^2^ = 0.051	*P* = 0.848 η_p_^2^ = 0.055

* Significance (*P* < 0.05).

### 3.2 Latency of SEPs

**FIGURE 4:**
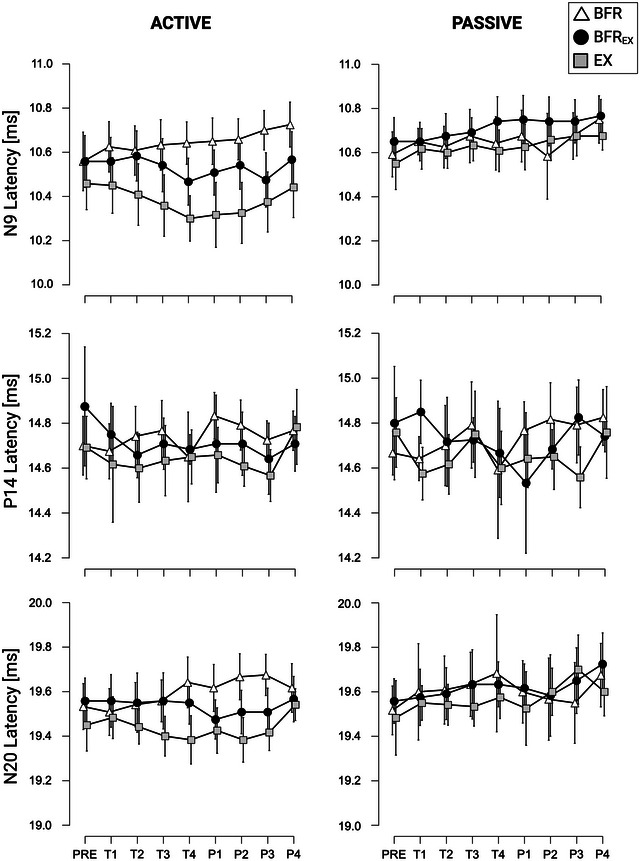
Mean somatosensory evoked potential (SEP) latencies (N9, P14 and N20) for ACTIVE and PASSIVE (in milliseconds). Mean values are indicated; the error bars represent 95% confidence intervals (*n* = 12 per condition). All individual responses can be found in the raw dataset provided.

With respect to N9 latencies, a significant effect for the factors CONDITION (*F*_2,22_ = 4.195, *P* = 0.029, η_p_^2^ = 0.276) and TIME (*F*_3.537,38.904_ = 3.304, *P* = 0.024, η_p_^2^ = 0.231) were found. Post‐hoc analyses revealed that latencies were reduced during EX (MD = −0.14 ms, SE = 0.05 ms, *P* = 0.038, *d* = −0.183) when compared with BFR (see Figure 4). In addition, a significant increase in latency was observed from PRE to P4 (MD = 0.09 ms, SE = 0.02 ms, *P* = 0.001, *d* = 0.118), T2 to P4 (MD = 0.07 ms, SE = 0.02 ms, *P* = 0.038, *d* = 0.09), T4 to P4 (MD = 0.09 ms, SE = 0.02 ms, *P* = 0.003, *d* = 0.111) and P2 to P4 (MD = 0.07 ms, SE = 0.02 ms, *P* = 0.046, *d* = 0.088). Additionally, a significant interaction effect of LIMB × CONDITION was found (*F*_2,22_ = 10.148, *P* = 0.0008, η_p_^2^ = 0.480). Simple main effects analysis for LIMB showed that N9 latencies were significantly lower in ACTIVE compared with PASSIVE during EX (*F*_1,11_ = 7.587, *P* = 0.019, η_p_^2^ = 0.408) but not during BFR_EX_ (*F*_1,11_ = 3.598, *P* = 0.084, η_p_^2^ = 0.246) and BFR (*F*_1,11_ = 0.008, *P* = 0.931, η_p_^2^ = 0.001). Furthermore, pairwise post‐hoc analyses indicated significantly reduced N9 latencies of ACTIVE during EX when compared with BFR (MD = −0.26 ms, SE = 0.06 ms, *P* = 0.002, *d* = −0.335). Last, a significant interaction effect of LIMB × CONDITION × TIME was revealed (*F*_16,176_ = 2.096, *P* = 0.010, η_p_^2^ = 0.256). Post‐hoc analyses indicated that N9 latencies of ACTIVE were significantly lower during EX compared with BFR at time points T4 (MD = −0.34 ms, SE = 0.07 ms, *P* = 0.020, *d* = −0.435), P1 (MD = −0.33 ms, SE = 0.07 ms, *P* = 0.030, *d* = −0.424), P2 (MD = −0.33 ms, SE = 0.07 ms, *P* = 0.030, *d* = −0.424) and P3 (MD = −0.33 ms, SE = 0.07 ms, *P* = 0.045, *d* = −0.414).

Concerning N20 latencies, a significant interaction effect of LIMB × CONDITION × TIME was revealed (*F*_16,176_ = 3.422, *P* < 0.0001, η_p_^2^ = 0.237). However, post‐hoc analyses failed to reach significance.

No further effects of LIMB, CONDITION, TIME, LIMB × CONDITION or LIMB × CONDITION × TIME were revealed for N9, P14 and N20 latencies. For exact statistics, please see Table 3.

**TABLE 3:** Results of repeated‐measures ANOVA investigating somatosensory evoked potential latencies for the factors LIMB, CONDITION and TIME and the interactions of LIMB × CONDITION and LIMB × CONDITION × TIME.

Component	LIMB	CONDITION	TIME	LIMB × CONDITION	LIMB × CONDITION × TIME
N9	*P* = 0.112 η_p_^2^ = 0.213	*P* = 0.029^*^ η_p_^2^ = 0.276	*P* = 0.024^*^ η_p_^2^ = 0.231	*P* = 0.0008^*^ η_p_^2^ = 0.480	*P* = 0.010^*^ η_p_^2^ = 0.160
P14	*P* = 0.815 η_p_^2^ = 0.001	*P* = 0.310 η_p_^2^ = 0.101	*P* = 0.204 η_p_^2^ = 0.113	*P* = 0.891 η_p_^2^ = 0.010	*P* = 0.628 η_p_^2^ = 0.072
N20	*P* = 0.129 η_p_^2^ = 0.197	*P* = 0.358 η_p_^2^ = 0.089	*P* = 0.095 η_p_^2^ = 0.183	*P* = 0.109 η_p_^2^ = 0.183	*P* < 0.0001* η_p_^2^ = 0.237

* Significance (*P* < 0.05).

### 3.3 EMG data

**FIGURE 5:**
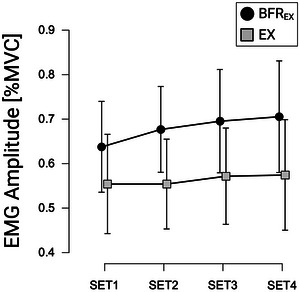
Mean EMG amplitudes of the biceps brachii muscle during resistance exercise for multiple sets with blood‐flow restriction (BFR_EX_) and without (EX) (expressed as a percentage of maximum voluntary contraction). Mean values are indicated; the error bars represent 95% confidence intervals (*n* = 12 per condition). All individual responses can be found in the raw dataset provided. Abbreviation: MVC, maximum voluntary contraction.

With respect to MVC‐normalized EMG amplitudes, no significant effect for CONDITION (*F*_1,11_ = 1.633, *P* = 0.228, η_p_^2^ = 0.129) or SET (*F*_1.683,18.515_ = 2.984, *P* = 0.083, η_p_^2^ = 0.213) and no significant interaction of CONDITION × SET (*F*_1.879,20.670_ = 0.903, *P* = 0.415, η_p_^2^ = 0.076) was found (see Figure 5). Pooled EMG amplitudes showed no significant difference (*t*_11_ = 1.278, *P* = 0.228, *d* = 0.369) between BFR_EX_ (67.9% MVC ± 36.9% MVC) and EX (56.4% MVC ± 23.8% MVC).

## 4 DISCUSSION

We aimed at examining the impact of BFR_EX_ on somatosensory processing, to assess the strain imposed on this system. Analysing amplitudes and latencies of short‐latency median nerve SEP, in addition to surface EMG activity of biceps brachii during various occlusive conditions, we observed predominately unaffected somatosensory processing. All findings and their implications are discussed in detail below.

Amplitudes of SEPs remained mostly unchanged throughout the conditions. The only instance of amplitude modulation we observed pertained to a main effect of the factor TIME in the P8–N9 component. Previous research demonstrated that acute exercise has an influence on SEP amplitudes, although these studies mainly investigated endurance exercise (Amjad et al., 2020). Nevertheless, one study showed that performing contractions of intrinsic hand muscles at different load levels (25% and 75% of maximum force) had an influence on the amplitude of SEPs (Chuang et al., 1999). The authors found differences in the degree of influence between various SEP components. Similar to the present study, N20–P25 amplitudes remained rather stable, whereas amplitudes of P8–N9 increased over time. Perspectively, it seems reasonable to investigate SEP characteristics during several load levels, in order to disentangle precisely the potential mechanisms of SEP amplitude modulation during BFR_EX_ protocols.

We also observed a significant decrease in N9 latency when comparing ACTIVE and PASSIVE during EX. This observation was to be expected, given previous research on the attenuating influence of acute exercise on SEP latencies (Nakata et al., 2016). Studies have shown a decrease in SEP latency for several components following submaximal bouts of cycling on an ergometer (Nakata et al., 2016; Perciavalle et al., 2015), in addition to submaximal treadmill exercise (Bulut et al., 2003). In contrast, a study by Amjad et al. (2020) failed to observe any changes in SEP latency after acute exercise. Notably, the authors attributed this divergence to their use of older patients while using a less demanding exercise protocol. A plausible explanation for the observed decrease of N9 latencies between ACTIVE and PASSIVE for EX in our study seems to be related to the strenuous nature of the exercise. Previous research was able to demonstrate interactions between decreased latencies of SEP components and (1) increases in blood lactate, an indicator for increased anaerobic metabolism, which is crucial to cover the short‐term high‐energy demands of peripheral musculature during high‐intensity exercise (Perciavalle et al., 2015), in addition to (2) increases in body temperature (Nakata et al., 2016). Although neither of these parameters was measured in the present study, it seems plausible that a similar link also applies to the present results. This assumption might be supported by the fact that the interaction between LIMB and CONDITION occurs only during the later measurement time points (T4–P3; i.e., after significant exercise), which could indicate an influence of either increasing metabolic demand or increasing body temperature on SEP latencies. Additionally, we observed decreased N9 latencies between EX and BFR for the ACTIVE but not for PASSIVE. As outlined above, increases in metabolic demand and body temperature during exercise can attenuate latencies of SEP. Notably, one might therefore expect that the N9 latencies between BFR_EX_ and BFR would also be reduced for ACTIVE. However, our results do not support such a conclusion. Instead, we argue that a second, opposing mechanism is responsible for the lack of a significant difference in N9 latencies between BFR_EX_ and BFR. It is known that prolonged nerve compression can reduce nerve conduction velocities (Kiernan & Bostock, 2000). Accordingly, the mechanical stress on nerve tissue following external compression can alter conduction properties and subsequently lead to increased SEP latencies (Jones et al., 2004). In this context, the absence of a difference in N9 latencies between BFR_EX_ and BFR, coupled with a significant decrease in N9 latencies in EX compared with BFR, can potentially be explained by the opposing effects of latency‐increasing nerve compression by the cuff (present in both BFR_EX_ and BFR conditions) and latency‐decreasing temperature increase and metabolic demand (present in both BFR_EX_ and EX conditions). However, this remains speculative and requires further investigation in future research to elucidate the precise mechanisms underlying these findings.

In general, factors such as temperature, anaesthesia, external compression and ischaemia are known to affect SEP amplitudes and latencies (Lang et al., 2002; Yang et al., 1995). Given that tourniquet‐induced compression leads to tissue hypoxia and ischaemia, the lack of systematic SEP modulation after BFR_EX_, BFR or EX conditions observed in the present study implies the absence of sustained peripheral ischaemia. This is corroborated by previous research attributing the brief effect of an inflated cuff on somatosensory processing to ischaemia rather than mechanical compression (Yamada et al., 1981). Peripheral ischaemia following arterial occlusion has been demonstrated to reduce or even completely suppress short‐latency SEP amplitudes and latencies (Hayashi et al., 2019; Jones et al., 2004; Yamada et al., 1981). Ischaemia disrupts the transmission of sensory signals along peripheral nerves, resulting in reduced afferent input to the central nervous system. This impairment can subsequently affect both the amplitude and latency of SEPs (Jones et al., 2004). Although it is known that BFR_EX_ can induce ischaemic conditions (Ferguson et al., 2018), the lack of systematic effects on SEP amplitudes observed in the present study could be related to differences in the duration of the ischaemic conditions and the occlusion pressure applied. In a preceding investigation, for example, peripheral ischaemia was induced by applying an occlusion pressure of 80 mmHg above systolic blood pressure, which was maintained for 30 min (Lopez‐Alburquerque et al., 1987). In the present study, all conditions were carried out using 50% of AOP, based on contemporary guidelines on BFR (Patterson et al., 2019), and this pressure was maintained for only 10 min. Furthermore, the pressure required completely to occlude arterial flow to a given limb in a seated position has been shown to range from 175 to 230 mmHg (Hughes et al., 2018). In contrast, the average tourniquet pressure applied in our study was 73.3 ± 8.2 and 74.5 ± 8.4 mmHg for BFR and BFR_EX_, respectively. Accordingly, the occlusive conditions chosen in our study were presumably insufficient to decrease blood flow sufficiently to cause sustained ischaemia. Interestingly, previous research on cuff occlusion applied to the arm suggests a non‐linear relationship between the percentage of arterial occlusion pressure (%AOP) and blood flow at rest. Specifically, these findings indicate that, at rest, lower pressures (up to ∼40% AOP), exhibit similar efficacy in restricting brachial arterial inflow when compared with higher pressures (∼80% AOP) (Mouser, Dankel et al., 2017). Crucially, this relationship was demonstrably altered in exercise conditions, in which significant differences in blood flow were observed between 40% and 80% AOP when performing four sets of biceps curls (Mouser, Laurentino et al., 2017). Another study showed that lowering cuff pressure from 80% AOP to more comfortable levels initially had negligible impact on blood flow until reaching a crucial threshold, typically falling within the 20%–40% AOP range (Mouser et al., 2018). Beyond this threshold, even slight reductions in the percentage of AOP resulted in substantial enhancements in brachial artery blood flow. It is therefore conceivable that the choice of %AOP dictates the impact of BFR_EX_ on SEP amplitudes and latencies. In general, BFR_EX_ should be performed between 40% and 80% of AOP, because higher pressure has been reported to induce discomfort in participants (Jessee et al., 2017). Furthermore, using the minimal pressure required to elicit a training response offers perceptual advantages, because it reduces stress for the individual undergoing training, which, in turn, promotes better adherence to exercise or therapeutic regimens (Vanwye et al., 2017).

Finally, EMG data also did not reveal significant differences between BFR_EX_ and EX. These findings appear to contradict a previous study, which demonstrated increased EMG activity during BFR_EX_ with low load levels compared with identical load levels without BFR_EX_ (Husmann et al., 2018). Despite using identical repetition schemes, notable distinctions existed between the protocols of the two studies regarding the specific exercises examined (knee extension vs. biceps curls), the duration of inter‐set resting periods (30 vs. 60 s) and the AOP during BFR_EX_ (60% vs. 50% AOP). Moreover, in the study by Husmann et al. (2018) all participants successfully completed the study protocol, whereas in our present study, 2 of 12 participants reached muscular failure during BFR_EX_. However, given that we did not ask the participants about their proximity to failure after each set, the number of participants who trained close to their individual capacity remains unclear. Previous research indicated that disparities in EMG activity between BFR_EX_ and corresponding conditions without BFR_EX_ diminish when muscular contractions are carried out to muscular failure, as was the case in some of the participants in our study (Cook et al., 2013). Thus, alongside the disparities in study protocols, the absence of differences in EMG activity between BFR_EX_ and EX might be attributed to the potentially higher levels of fatigue experienced by our sample population. Future studies should explore more thoroughly how peripheral and central fatigue, as in addition to different levels of mechanical tension, affect muscle activity during BFR_EX_ to gain a clearer understanding of sensorimotor processing in occlusive exercise conditions (Centner & Lauber, 2020).

### 4.1 Limitations

Fundamentally, BFR_EX_ is a method that is still based on individual assessments. For instance, there are various guidelines and practices for determining the AOP, the choice of cuff width and the cuff material (Patterson et al., 2019). The AOP depends on the width of the cuff, with a wider cuff requiring less pressure to stop blood flow to a given limb (Jessee et al., 2016). Thus, the inclusion of different cuff sizes would have been a viable addition. Nonetheless, previous studies have shown that the specification of pressure as %AOP, as in the present study, led to comparable changes in blood flow with three different cuff sizes (Mouser, Dankel et al., 2017). Furthermore, based on the previously outlined findings, it will be interesting to study the potential effects of different load levels during BFR_EX_ on somatosensory processing and associated modulations in SEP amplitude and latency. Given that BFR_EX_ is generally performed using load levels between 15% and 50% of the maximum force, the scope of SEP modulation induced by BFR_EX_ must be extended to several load levels in order to increase ecological validity. With our results, we provide an initial contribution. However, based on previous research (Chuang et al., 1999), it cannot be ruled out that unique modulation might occur, depending on specific load levels.

Another point for consideration centres on the potential for global cardiovascular and somatosensory modulations induced by unilateral BFR_EX_ and EX conditions, which might affect contralateral and passive limbs. Importantly, local ischaemia, the primary mechanism assumed to drive adaptations following BFR_EX_, is limited to the trained limb. Peripheral nerve compression, another potential modulator of SEP characteristics, also did not affect the passive limb. On the contrary, central nervous cross‐wiring enables potential affection of contralateral, passive limbs following unilateral contractions. Crucially, our research findings reveal minimal alterations in SEP characteristics for the passive limb following both BFR_EX_ and traditional training conditions. Thus, although global effects might accompany unilateral contractions, they do not systematically influence the somatosensory parameters investigated in our study. Further research should incorporate thorough assessments of bilateral cardiovascular and neuromuscular functioning to advance the understanding of such potential effects. Still, the absence of passive control conditions without cuff application or unilateral resistance exercise is a limitation that should be addressed in future studies to rule out any transfer effects from the trained to the untrained limb during unilateral contractions.

Another aspect for consideration pertains to the time point of SEP measurements. Given that SEPs are usually recorded before and/or after motor performance owing to methodological limitations, our results concern only those time points and do not shed light on the modulation of SEP components during the performance of unilateral resistance exercise. Finally, in order to disentangle modulatory distinctions relating to SEP components of different origins, multimodal studies incorporating measures of peripheral and cortical excitability, such as SEPs and motor evoked potentials, will be beneficial in future studies.

## 5 CONCLUSION

In conclusion, our study provides evidence for the impact of acute BFR_EX_ within the somatosensory domain of healthy adults, according to current guidelines. It is crucial to identify potential risks associated with BFR_EX_ in advance to ensure safe use in different populations. Our results show minimal effects on nervous system function, particularly on somatosensory processing, adding to the available evidence concerning the application of BFR_EX_ (Mendonca et al., 2020). Future research should investigate load‐dependent modulations of somatosensory processing using multimodal approaches to refine BFR_EX_ protocols. Given the intended implementation of BFR_EX_ in rehabilitative settings, future research should aim to replicate our findings in patients to strengthen our conclusion. Overall, BFR_EX_ proves to be a promising alternative to conventional high‐load resistance exercise, with potential benefits in musculoskeletal and neurological rehabilitation.

## AUTHOR CONTRIBUTIONS

Tom Maudrich, Rouven Kenville and Patrick Ragert designed the study. Tom Maudrich and Sebastian Degener acquired the data. Tom Maudrich, Sebastian Degener and Rouven Kenville analysed the data and wrote the manuscript. Patrick Ragert provided critical revision. All authors interpreted the data, contributed to the manuscript, reviewed it, approved the content of the final version and agreed to be accountable for all aspects of the work in ensuring that questions related to the accuracy or integrity of any part of the work are appropriately investigated and resolved. All persons designated as authors qualify for authorship, and all those who qualify for authorship are listed.

## CONFLICT OF INTEREST

None declared.

## FUNDING INFORMATION

None.

## Data Availability

Data, in an anonymous format (according to data protection policy in the ethics agreement), are available at https://figshare.com/articles/dataset/Data_BFR_SEP_xlsx/25348465
